# Cephalexin-Induced Hemolytic Anemia and Renal Failure: A Case Report and Review of the Literature

**DOI:** 10.7759/cureus.96370

**Published:** 2025-11-08

**Authors:** Swati Gobhil, Arjun Khunger

**Affiliations:** 1 Department of Hospital Medicine, Sacred Heart Medical Center, Springfield, USA

**Keywords:** acute renal failure, acute tubular necrosis (atn), adverse drug events, antibiotic-induced renal failure, cephalexin, cephalexin-induced renal failure, drug-induced hemolysis

## Abstract

Hemolytic anemia is a rare adverse reaction to cephalosporins, and its occurrence in response to cephalexin is even rarer. Cephalexin is a commonly prescribed empiric oral antibiotic for a variety of infections, particularly in the outpatient setting. It is, therefore, important to be aware of this possible adverse reaction to cephalexin for timely intervention and prompt discontinuation of the offending agent. We present a case of a 48-year-old female with no significant past medical history who presented with intermittent hematuria for two days and anuria for one day. She had been seen in the emergency department three days prior for a urinary tract infection and discharged on cephalexin. Her renal function was normal at the time. Upon re-presentation with anuria, her creatinine and hemoglobin had acutely worsened, lactate dehydrogenase was elevated, and haptoglobin was low. Her renal function rapidly deteriorated, and the patient required hemodialysis. Her renal biopsy revealed acute tubular injury with numerous intraluminal hemoglobin casts supporting the diagnosis of drug-induced hemolysis resulting in acute renal failure. The patient achieved full renal recovery within four weeks following prompt discontinuation of cephalexin and timely renal replacement support. This case highlights the importance of considering this rare adverse reaction to cephalexin when a patient presents with findings suggestive of intrarenal acute kidney injury. Early recognition can expedite the diagnostic workup and treatment, potentially improving outcomes.

## Introduction

Cephalexin is one of the most commonly prescribed antibiotics for infections such as skin infections, cystitis, joint infections, and pharyngitis. Hemolytic anemia is an uncommon adverse reaction of cephalosporins, and is particularly rare with cephalexin. Drug-induced hemolysis occurs when certain medications trigger immune-mediated destruction of red blood cells. This can happen through various mechanisms, which can involve hapten formation, autoantibody production, or oxidative stress [1-3]. The resulting intravascular hemolysis releases free hemoglobin into the circulation. In high concentration, free hemoglobin is toxic to renal tubular epithelial cells, leading to oxidative injury, tubular obstruction by hemoglobin casts, and ischemia. This can lead to acute tubular necrosis characterized by impaired renal function, and, in severe cases, anuria, prompting the need for renal replacement therapy at times [4]. Only a few case reports have been published over the past four decades describing this adverse reaction. We present one such case, along with a literature review of other cases with similar findings.

## Case presentation

We present the case of a generally healthy 48-year-old female with no significant past medical history who presented to the emergency room with intermittent hematuria for two days and minimal urine output for one day. The patient was seen in the emergency department three days prior to this visit for increased urinary frequency, incomplete bladder emptying, and low back pain. At that time, her white blood cell (WBC) count was normal at 9200/mcL, hemoglobin was low at 8.9 g/dL, and platelet count was normal at 247,000/mcL. Creatinine was normal at 0.54 mg/dL. Her urine analysis (UA) revealed 3+ blood, pyuria of 15-20 WBC/high power field (hpf), microscopic hematuria with 61-100 RBC/hpf, and nitrite and leukocyte esterase could not be determined due to color interference. A computed tomography (CT) of the abdomen and pelvis without contrast was obtained and was negative for urolithiasis or hydronephrosis. The patient was prescribed cephalexin 500 mg twice daily for presumed cystitis.

Upon re-presentation, the patient reported intermittent hematuria for two days and only a few drops of dark red urine for one day. She denied any dysuria, lower abdominal pain, fever, chills, nausea, vomiting, or blood in stool. The patient denied use of any non-steroidal anti-inflammatory drugs (NSAIDs). She denied taking any other over-the-counter medications. Her only medication was the prescribed cephalexin. On clinical examination, her vital signs were stable, and her physical exam was unremarkable. She was euvolemic at the time of admission. The patient was found to have elevated creatinine from 0.54 mg/dL three days prior to 5.62 mg/dL. Her hemoglobin had acutely worsened and dropped from 8.9 g/dL to 6.4g/dL. Her lactate dehydrogenase (LDH) was significantly elevated, and haptoglobin was undetectable, raising suspicion for hemolysis. The patient's relevant lab results on presentation are outlined in Table 1.

**Table 1:** Relevant bloodwork on admission. BUN: blood urea nitrogen; eGFR: estimated glomerular filtration rate; RBC: red blood cells; CK: creatine kinase; LDH: lactate dehydrogenase; TSAT: transferrin saturation; INR: international normalized ratio.

Test	Lab value	Reference range
Creatinine	5.62	0.55-1.02 mg/dL
BUN	62	9-23 mg/dL
eGFR	9	>=60 mL/min/1.73 m^2^
RBC	3.35	3.75-5.07 M/uL
Hemoglobin	6.4	11.5-15.0 gm/dL
Platelet	206	150-400 K/uL
CK	50	38-234 U/L
LDH	1146	120-246 U/L
Ferritin	206.3	7.0-271.0 ng/mL
Iron	27	28-170 mcg/dL
TSAT	8	15-50%
Reticulocyte %	2.8	0.4-2.5%
Immature retic fraction	19.6	1.9-15.1%
INR	1.0	0.9-1.1
Haptoglobin	<10	30-200 mg/dL
Vitamin B12	196	211-911 pg/mL
Folate	14.0	>5.4 ng/mL
Total bilirubin	0.5	0.3-1.2 mg/dL
Indirect bilirubin	0.2	<0.3 mg/dL

The renal ultrasound on admission was negative for hydronephrosis, and it revealed a decompressed urinary bladder. A renal artery duplex was negative for any hemodynamically significant stenosis within the renal arteries bilaterally.

**Figure 1:**
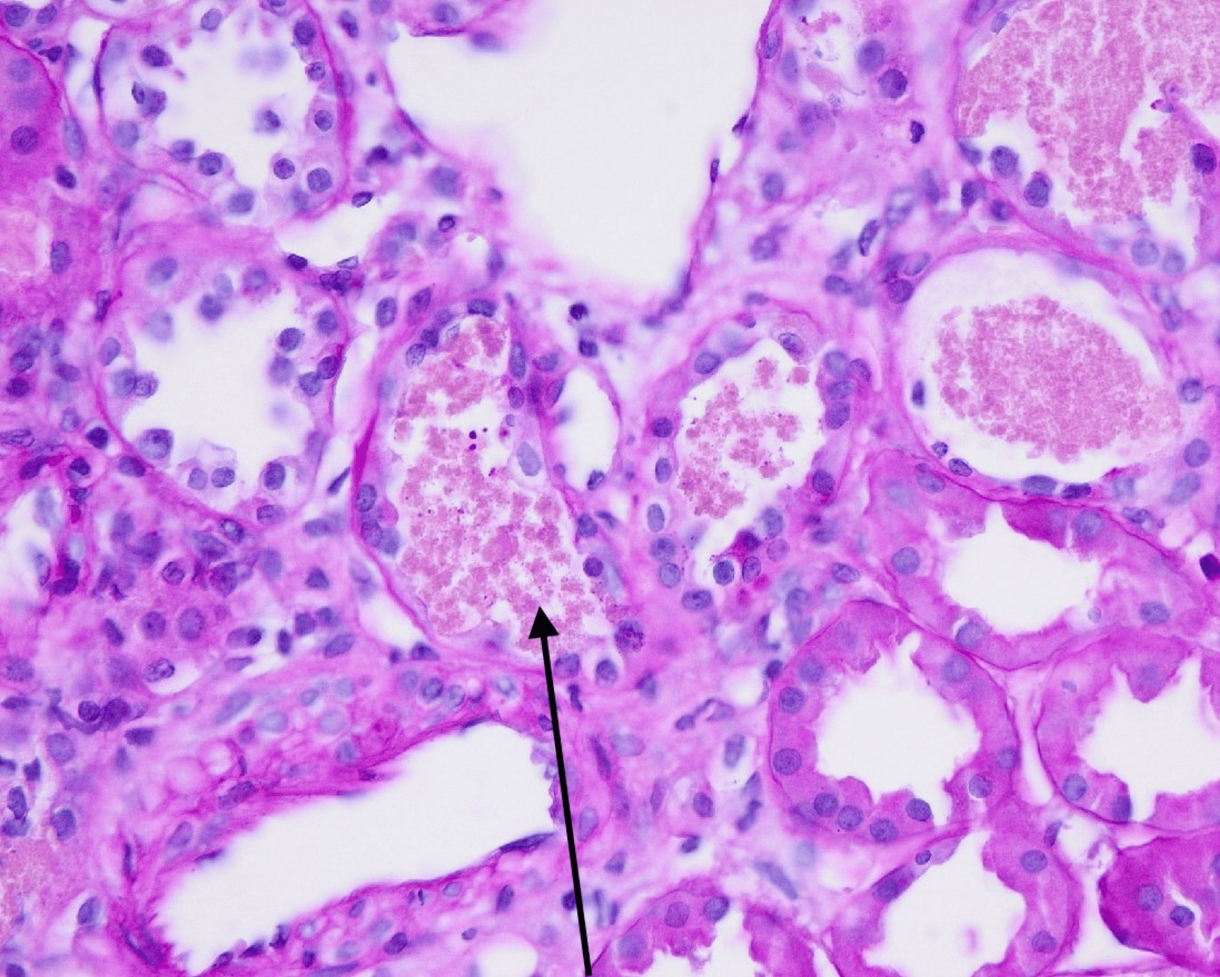
This picture demonstrates pigmented hemoglobin casts inside renal tubules.

**Figure 2:**
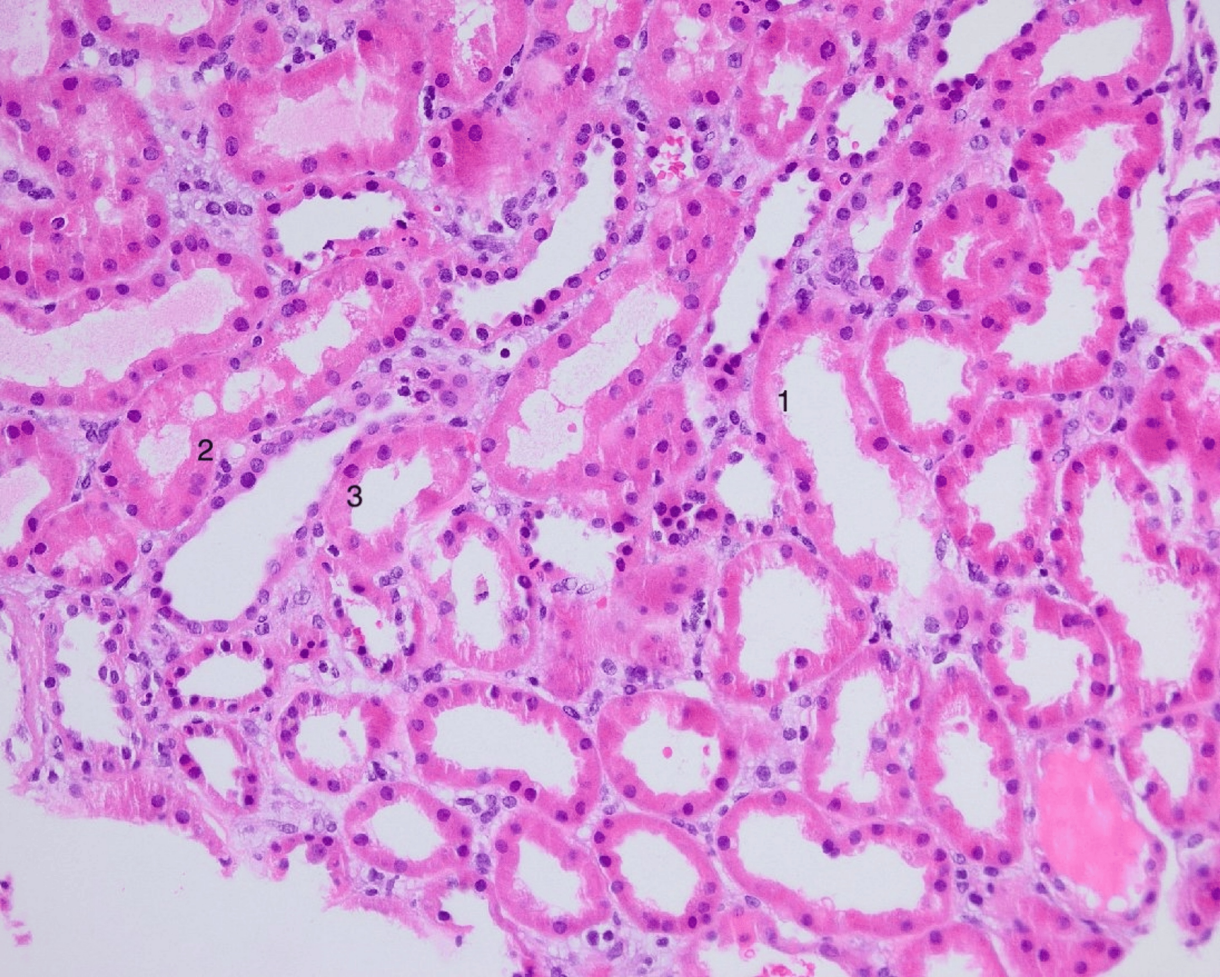
This histopathology picture demonstrates epithelial line flattening (1), epithelial lucency (2), and tubular nuclei loss (3), all of which point toward acute tubular injury.

Antineutrophil cytoplasmic antibody, anti-proteinase 3 (anti-PR-3) antibody, and anti-myeloperoxidase (anti-MPO) antibody returned negative. Antibodies to nuclear antigen were positive. Hep 2 cytoplasmic pattern was noted (cytoplasmic speckled pattern, which is often associated with antibodies to ribosomal RNA synthetase, such as Jo-1 and related myositis). Her direct antiglobulin test (DAT) remained consistently negative throughout the hospitalization. No abnormally elevated schistocyte count was noted. Infectious work-up, including acute hepatitis panel and leptospira DNA PCR, was negative. Urine and blood cultures were negative. The cold agglutinin assay was negative. Glucose-6-phosphate dehydrogenase assay revealed elevated levels. Hemoglobinopathy/thalassemia evaluation revealed a normal hemoglobin phenotype. Peripheral blood smear revealed severe microcytic hypochromic anemia with no significant increase in schistocytes. Bone marrow biopsy revealed hypercellular bone marrow (70%) with predominant erythroid hyperplasia. A renal biopsy was promptly pursued in the setting of rapidly worsening renal function, and it revealed acute tubular injury with numerous intraluminal hemoglobin casts, as demonstrated below in Figures 1, 2.

The patient was started on methylprednisolone on admission. A hemodialysis catheter was placed within two days of admission, and the patient was started on hemodialysis. Eventually, her workup was felt to be most consistent with cephalexin-induced hemolysis leading to acute tubular injury. The prednisone was gradually tapered over the next few weeks. Her anemia was felt to be secondary to iron deficiency at baseline, which had acutely worsened due to hemolysis. The patient was discharged home with a plan to continue outpatient hemodialysis eight days later. She required hemodialysis for about three weeks total, and subsequently had complete renal function recovery.

## Discussion

Drug-induced hemolytic anemia (DIHA) is a rare but potentially serious complication of drug administration with an estimated incidence of 1-2 per million cases per year [5]. Antibiotics and chemotherapy drugs are most frequently associated with DIHA. Among antibiotics, the second- and third-generation cephalosporins have been found to be leading causes of DIHA [5]. In contrast, first-generation cephalosporins (e.g., cephalexin, cephalothin, and cefazolin) are rarely associated with DIHA [6]. DIHA is predominantly an immune-mediated process that results in intravascular hemolysis. It can be associated with severe complications such as renal failure, hepatic dysfunction, and disseminated intravascular coagulation. Intravascular hemolysis in DIHA releases free hemoglobin, which is toxic to renal tubular cells, leading to acute kidney injury (AKI) or renal failure [7]. Several mechanisms of DIHA have been identified in association with cephalosporin use. It includes immune complex formation, hapten-mediated reactions, autoantibody production, or direct oxidative injury to erythrocytes [8]. Given the diverse mechanisms, immune marker assays, such as the DAT, may yield positive or negative results depending on the specific pathway involved. DAT detects antibodies on RBCs, and it is used as a screening test to detect the same, which helps differentiate immune from non-immune hemolysis [9]. Often, hemolysis due to non-immune-mediated pathways or low-affinity antibodies may result in a negative DAT test, complicating diagnosis [2,9].

In our patient, there was evidence of acute hemolysis, as evidenced by an acute drop in hemoglobin from 8.9 g/dL to 6.4 g/dL in three days, increased reticulocyte count, increased LDH of 1146 U/L, and low haptoglobin of <10 mg/dL. Subsequently, the renal biopsy revealed hemoglobin-laden tubules accompanied by acute tubular necrosis. Antinuclear antibody (ANA) was positive with Hep2 cytoplasmic pattern, but felt to be a false positive with negative anti-double-stranded DNA (anti-dsDNA). Antineutrophil cytoplasmic antibody (ANCA) was negative, and complement levels were normal. We could not identify any specific cause of hemolytic anemia, hence supporting our diagnosis of DIHA. Given the patient’s young age, absence of comorbidities with no concomitant medication or herbal supplement use, and recent administration of cephalexin for a urinary tract infection, cephalexin was considered the most likely etiology of her DIHA. The DAT was done multiple times during hospitalization, including a super Coombs test (enhanced DAT), and the results were consistently negative. Enhanced DAT is a more sensitive version of DAT, which can detect antibodies even at low levels or with lower affinity than standard DAT [9]. The negative DAT test observed in our patient may be attributed to the sudden onset of massive hemolysis caused by cephalexin, and therefore the low levels of sensitized RBC with C3d and IgG at the time of presentation. This has been previously described in ceftriaxone-induced immune hemolytic anemia [8]. Additionally, the subsequent decline in LDH levels showed that the hemolysis was transient and ceased after discontinuation of the offending drug. Further, the presence of hemoglobin casts in the renal biopsy supports the notion of massive transient hemolysis likely initiated by an acute inciting event such as drug exposure [8].

Our literature review identified multiple prior reports of cephalexin-induced hemolytic anemia, which have been summarized in Table 2 [10-14]. A few cases had patients with prior penicillin allergy, suggesting that cephalexin-induced hemolytic anemia could be due to cross-reactivity between penicillin and cephalosporins, which share a beta-lactam ring structure that may trigger an immune response in sensitized individuals [10-14]. Such cases also had positive DAT on testing. Our patient did not have any known prior penicillin allergy and hence was less likely to have an immune complex or complement-mediated reaction, as suggested by negative DAT and normal complement levels. Similar to our case, renal failure secondary to hemolysis was severe, often necessitating hemodialysis. Renal biopsy findings were comparable, demonstrating evidence of cast nephropathy. Notably, the majority of patients showed resolution of both hemolysis and renal failure following discontinuation of the offending drug.

**Table 2:** Literature review of previously published case reports of cephalexin-induced hemolytic anemia. DAT: direct antiglobulin test; AKI: acute kidney injury.

Author year	Presentation	Prior known penicillin sensitivity	DAT result	Presence of renal dysfunction	Outcome
Longstreth et al. (2004) [10]	A 24-year-old female with acute pharyngitis with a positive rapid streptococcal screen test, prescribed cephalexin	Yes	Unknown	AKI present, non-oliguric, hemodialysis not started. Electron microscopy revealed resolving tubular injury consistent with nephrotoxic acute tubular necrosis	Complete resolution
Baradhi et al. (2015) [11]	A 61-year-old female with acute bronchitis treated with prednisone and cephalexin	No	Positive DAT for immunoglobulin G and C3, negative complement levels	Required hemodialysis. Renal biopsy revealed pigment nephropathy	Unknown
Thiessen et al. (2017) [12]	A 44-year-old female given cephalexin for pan-sensitive Klebsiella pneumoniae infection	Yes	DAT negative	No	Complete resolution
Poloni et al. (2020) [13]	A 15-year-old female receiving cephalexin for a finger infection	Unknown	Unknown	Requiring hemodialysis. A renal biopsy was performed, which revealed numerous iron deposits (hemosiderin granules) within the casts	Complete resolution. Empiric steroids given
Markley et al. (2025) [14]	A 50-year-old female given cephalexin 2,000 mg by mouth for prophylaxis prior to a dental procedure	Unknown	DAT was positive for both IgG and anti-C3	Required hemodialysis. Kidney biopsy was performed and revealed hemoglobin cast nephropathy consistent with intravascular hemolysis	Complete resolution
Our case	A 48-year-old female received cephalexin for a UTI	None	Negative	Requiring hemodialysis. Renal biopsy was performed, which revealed acute tubular injury with intratubular hemoglobin casts	Complete resolution in 4 weeks

The most effective treatment for patients who develop DIHA is immediate discontinuation of the causative drug [15]. Cessation of hemolysis promotes hematological recovery and renal function, often within one to two weeks [15]. Our patient had a complete recovery as evidenced by hemoglobin of 10.8 and creatinine of 0.55 on outpatient follow-up in one month. The patient was taken off hemodialysis after three weeks and had full renal recovery in four weeks post hospitalization.

The role of glucocorticoids for the treatment of DIHA is not well defined. Often, oral prednisone with a dose of 1 mg/kg or 60-100 mg daily is administered, especially in severe cases [16]. In our patient, methylprednisolone 500 mg twice daily was started on day one for three days, followed by prednisone 60 mg by mouth daily, which was tapered over 10 weeks. However, since the suspected drug is withdrawn simultaneously with the administration of glucocorticoids, the beneﬁt of prednisone is difficult to prove. Therefore, the decision to use steroids should be individualized based on the severity of hemolysis and the patient’s overall clinical condition [16].

## Conclusions

Though rare, cephalexin can cause both immune-mediated and non-immune-mediated forms of DIHA. Given the widespread use of cephalexin to treat common infections, clinicians should be aware of this rare adverse effect when prescribing the same. In patients presenting signs of hemolytic anemia or acute renal failure, DIHA should be considered in the differential diagnosis. Management includes prompt discontinuation and future avoidance of the drug, and supportive therapies such as hemodialysis for renal failure. The overall prognosis is favorable, with most patients demonstrating good recovery.
